# A randomized, prospective clinical study evaluating effectiveness of a bulk-fill composite resin, a conventional composite resin and a reinforced glass ionomer in Class II cavities: one-year results

**DOI:** 10.1590/1678-7757-2018-0678

**Published:** 2019-10-07

**Authors:** Hacer Balkaya, Soley Arslan, Kanşad Pala

**Affiliations:** 1 Erciyes University, Faculty of Dentistry, Department of Restorative Dentistry, Kayseri, Turkey.

**Keywords:** Clinical trial, Composite resins, Glass ionomer cements

## Abstract

Bulk-fill restorative materials such as bulk-fill composite resins and high viscous glass ionomer cements have become very popular materials in operative dentistry because their application is easy and time-saving.

**Objectives:**

The aim of this clinical study was to evaluate the clinical performance of a highly viscous reinforced glass ionomer material, a bulk-fill composite resin and a micro hybrid composite resin in Class II restorations.

**Methodology:**

In total, 109 Class II restorations were performed in 54 patients using three different restorative materials: Charisma Smart Composite (CSC); Filtek Bulk Fill Posterior Restorative (FBF); Equia Forte Fil (EF). Single Bond Universal adhesive (3M ESPE, Germany) was used with composite resin restorations. The restorations were evaluated using modified USPHS criteria in terms of retention, color match, marginal discoloration, anatomic form, contact point, marginal adaptation, secondary caries, postoperative sensitivity and surface texture. The data were analyzed using Chi-Square, Fischer's and McNemar's tests.

**Results:**

At the end of one year, 103 restorations were followed up. No changes were observed during the first 6 months. At the end of one year, there were small changes in composite restorations (FBF and CSC) but no statistically significant difference was observed between the clinical performances of these materials for all criteria (p>0.05). However, there was a statistically significant difference between EF, FBF and CSC groups in all parameters except marginal discoloration, secondary caries and postoperative sensitivity in one-year evaluation (p<0.05).

**Conclusion:**

Bulk-fill composite resins and conventional composite resins showed more successful clinical performance than highly viscous reinforced glass ionomers in Class II cavities.

## Introduction

In modern dental practice, the advances in adhesive techniques, allied to increased focus on the aesthetic qualities of dental restorations and adoption of a minimally invasive dentistry approach, have great influence on the treatment plan in the posterior and anterior regions.^1^,^2^

In recent years, posterior composites have been generally preferred for back tooth restorations by direct methods because of their advantages such as single visit and short application time, aesthetics, ability to protect dental tissues during preparation, and being cheaper when compared to indirect methods.^3^ Negative results such as poor marginal adaptation, marginal discoloration, white line formation around the restoration, tubercle fractures, microleakage, secondary caries and postoperative sensitivity in composite resin restorations are generally based on polymerization shrinkage stress;^4^–^6^ thus, various attempts have been made to achieve low polymerization shrinkage in restorative materials.^7^ In addition, time-saving applications for the implementation of posterior restorations are in high demand. Almost a decade ago, resin-based bulk-fill composites were introduced to the market. High viscosity bulk-fill composites can be polymerized at 4 or 5 mm thickness in a single step, thus not requiring time-consuming layering. The main reasons why thick layering cannot be achieved in conventional resin composites are the limited depth of cure and the increase in polymerization shrinkage at the interface between tooth and restorative material.^8^,^9^ However, it has been reported that bulk-fill composites do not adversely affect the polymerization shrinkage, the adaptation of the cavities and the degree of conversion during application, and exhibit less polymerization shrinkage than conventional composite resins.^9^

In addition, glass ionomer cements (GIC) are materials that can be used as an alternative to composite resins in conservative restoration of caries lesions in the posterior region. GIC have advantages such as having a similar thermal expansion coefficient to natural tooth tissue, physicochemical adhesion to tooth tissues, fluoride release, biocompatibility, low shrinkage, low marginal leakage, anti-caries properties on the restoration edges, and increased remineralization in adjacent proximal caries.^10^,^11^ However, conventional GIC have disadvantages such as low fracture and abrasion resistance, inadequate color stability, moisture sensitivity and poor aesthetic properties. These disadvantages weaken the physical properties of the material and restrict its use in areas exposed to intensive chewing forces.^12^ In recent years, to reduce moisture sensitivity of GIC in early stages of hardening, to increase their hardness and abrasion resistance, and to enable them to be used in areas exposed to chewing forces, the materials were strengthened by changing the powder/liquid ratio, particle size and distribution, and highly viscous glass ionomer cements (HVGIC) were presented to the market.^12^ The hardening mechanisms of these newly developed HVGIC are the same as conventional GIC. Besides, abrasion resistance, fracture toughness, flexural strength, and sensitivity to moisture are improved when compared to conventional GIC.^13^ Moreover, early water exposure does not adversely affect the physical properties of these materials since the setting reaction is faster in HVGIC, unlike in conventional GIC.^14^ Nonetheless, the manufacturer suggests that these materials should be applied with surface coating resins.^15^ In 2007, a new HVGIC restorative system (EQUIA; GC Europe, Tokyo, Japan) was introduced, which could be an alternative to composite resins in the posterior region, and was designed for the use in the permanent restoration of Class I, II and V cavities by combining the advantages of HVGIC and a surface coating resin.^13^,^16^

The application of surface coating resins to GIC surface enhances the surface brightness of the material, prevents the translucency reduction of the material over time, fills the gaps caused by the material and finishing processes and surface irregularities to provide a smooth surface, reduces moisture sensitivity in the early stages of hardening, increases the resistance to fracture and abrasion, and improves mechanical properties.^15^ Today, there are new restorative surface coating agents reinforced by the addition of nano-fillers, low molecular weight monomers, photoinitiators and other variables.

The aim of this study was to evaluate the clinical performance of Class II restorations made with different restorative materials using modified United States Public Health Service (USPHS) criteria. The null hypothesis of this study was that there would be no difference between the one-year clinical performance of composite resin materials (conventional and bulk-fill) and HVGIC in Class II restorations.

## Methodology

**Figure 1:**
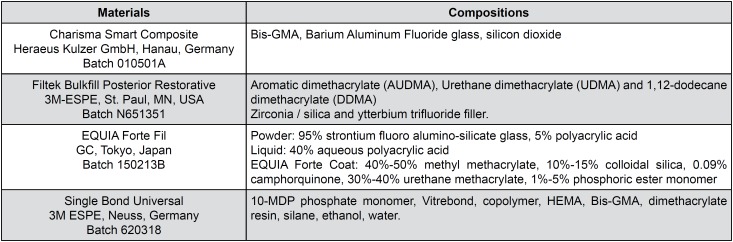
Materials, compositions and batch numbers

Before conducting the study, the research protocol was approved by the Faculty of Medicine Ethics Committee (Approval Number: 2017/44) at Erciyes University. In this randomized controlled clinical study, a HVGIC (Equia Forte Fil, GC, Tokyo, Japan), a bulk-fill composite resin (Filtek Bulk Fill Posterior Restorative, 3M ESPE, St. Paul, USA) and a micro hybrid composite resin (Charisma Smart Composite, Heraeus Kulzer, Hanau, Germany) were compared. These materials, compositions and batch numbers are given in Figure 1.

### Study Design and Patient Selection

**Figure 2:**
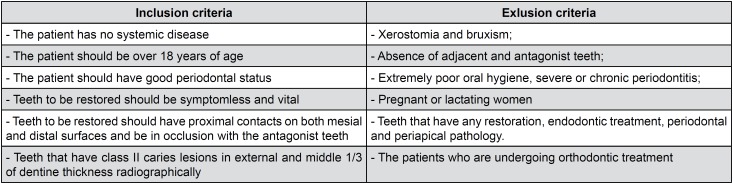
Inclusion and exlusion criteria

Patients attending the Department of Restorative Dentistry, Faculty of Dentistry, at Erciyes University for routine dental care were examined clinically and radiographically with bite-wing radiography. In this study, 80 patients were assessed for eligibility for participation, and 26 patients were excluded due to either failing to meet the inclusion criteria or declining to come for follow-up visits. In total, 54 patients who met the inclusion criteria were selected. The inclusion and exclusion criteria for the selection of patients for the study are shown in Figure 2. The volunteers participating in the study were informed about the research protocol and possible complications. Finally, an informed consent form was read and signed by the patients.

### Restorative Procedures

In this study, 109 teeth in 54 patients (31 female, 23 male) were randomly restored by an experienced dentist using the different restorative materials. The randomization of the restorative materials was done using a table of random numbers. The mean age of the patients was 22.25±2.5 years (range: 20-32 years). Initial bite-wing radiographs of the teeth to be treated were taken before the treatment. The most appropriate material color was selected before restorative procedures began. Local anesthesia was applied to patients complaining about pain or sensitivity to prevent discomfort during restorative procedures. Cavity preparations were performed using diamond fissure burs (Diamir srl, Resia, Italy) at high speed with water-cooling. Hand instruments and slow-speed tungsten carbide burs were used to remove the caries. Conservative cavity design (Class II slot) was used and bevelling was not applied to the cavity walls to avoid unnecessary loss of hard dental tissue. The cavity preparations did not involve any cusps, all the gingival margins included sound enamel, and two surfaces cavities (MO or DO) were included in this study. The outline shape of the cavity was limited to the removal of caries lesion. Any additional retention was not prepared. The depth of cavities was approximately 4-5 mm from the gingival border of the cavity when mesial or distal marginal ridge was taken as reference. Ca(OH)_2_ cavity liner material (Dycal, Dentsply, Konstanz, Germany) was applied where needed as base material (only for two restorations; one high viscosity glass ionomer and one conventional composite resin). Cotton pellets and suctions were used to isolate the operative field. After an ivory type matrix system (Hahnenkratt, Königsbach-Stein, Germany) and wooden wedges were placed on the cavities, they were disinfected with 0.2% chlorhexidine gluconate. All the cavities were restored as follows:

Group 1: Single Bond Universal adhesive (3M ESPE, Neuss, Germany) was applied to the cavities according to the manufacturer's instructions and polymerized with a LED light device (Valo, 1000 mW/cm^2^, Ultradent, Utah, USA) for 10 s. Charisma Smart Composite (CSC) was placed incrementally by using horizontal increments, not exceeding 2 mm, in the cavity and each layer was cured for 20 s. After removal of the matrix and wedges, the restorations were cured for additional 10 s from buccal and palatal/lingual sides.

Group 2: Single Bond Universal adhesive was applied and polymerized as in Group 1. Filtek Bulk Fill Posterior Restorative (FBF) was placed in bulk to the cavity to be no more than 4 mm thick, and was cured for 20 s. After removal of the matrix and wedges, the restorations were cured for additional 10 s from buccal and palatal/lingual sides.

Group 3: Cavity Conditioner (GC) was applied to the cavities for 10 s, washed, and gently dried. After isolation, an Equia Forte Fil (EF) capsule was placed in an automatic mixer and stirred for 10 s. The capsule was then placed in a special applicator and injected into the cavities. After the manufacturer's recommended setting time of 2.5 minutes, the restoration was finished, polished and gently dried. Equia Forte Coat (GC) was applied to the restoration surfaces and cured for 20 s.

Finishing and polishing procedures were performed in the same appointment using high-speed fine diamond burs (Meisinger Dental Burs, Hager & Meisinger GmbH, Neuss, Germany), Sof-Lex XT discs (3M ESPE, St. Paul, USA) and yellow composite polishing rubbers (Nais, Sofia, Bulgaria).

### Clinical Evaluations of the Restorations

**Figure 3:**
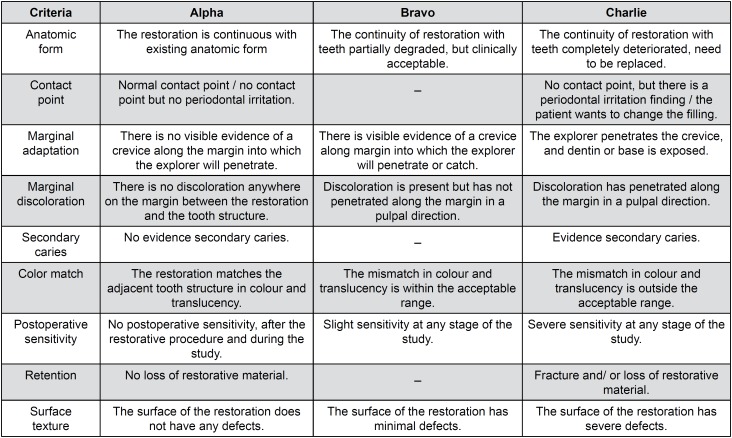
Modified United States Public Health Service (USPHS) criteria used in this study

In this study, all the participating dentists were trained for calibration before the study conduction. After restoration placement, patients were followed-up after one week (baseline), six months and one year. The restorations were clinically examined using mirrors and probes, and bite-wing radiographs and intraoral photographs were taken from the patients. The restorations were evaluated by two experienced double-blinded dentists according to modified USPHS criteria (Figure 3), in terms of anatomic form, contact point, color match, marginal discoloration, marginal adaptation, surface texture, secondary caries, postoperative sensitivity and retention. When any disagreement occurred during the evaluation, the final decision was made by a consensus of both evaluators.

### Statistical Analysis

The information obtained was collected in a data pool and statistical analyses were performed using the software SPSS 22.0 (SPSS; Chicago, IL, USA). Frequency and rate values were used in descriptive statistics of the data. Chi-square test and Fischer's test were used in the analysis of independent qualitative data. Moreover, McNemar's test was used for the analysis of the dependent qualitative data.

## Results

In total, 109 restorations were placed in 54 patients and, with a 95% recall rate, 103 restorations were evaluated at 6-month and one-year recall. Regarding the teeth, 46 restorations (44.7%) were placed in premolars, whereas 57 (55.3%) were placed in molars. Clinical evaluation scores of restorations at baseline, 6-months and one year are given in Table 1. There was no significant difference between premolar and molar teeth for all parameters and all evaluation periods (p>0.05).

**Table 1:** Baseline, six-month and one-year clinical evaluation of restorations according to USPHS criteria (%)

CRITERIA	BASELINE			6-MONTH			ONE-YEAR		
	**A**	**B**	**C**	**A**	**B**	**C**	**A**	**B**	**C**
**Anatomic Form**									
CSC	35 (100)	0	0	35 (100)	0	0	35 (100)	0	0
FBF	36 (100)	0	0	36 (100)	0	0	36 (100)	0	0
EF	32 (100)	0	0	32 (100)	0	0	26 (81,25)	4 (12,5)	2 (6,25)
**Contact point**									
CSC	35 (100)	–	0	35 (100)	–	0	35 (100)	–	0
FBF	36 (100)	–	0	36 (100)	–	0	36 (100)	–	0
EF	32 (100)	–	0	32 (100)	–	0	27 (84,4)	–	5 (15,6)
**Marginal adaptation**									
CSC	35 (100)	0	0	35 (100)	0	0	30 (85,7)	5 (14,3)	0
FBF	36 (100)	0	0	36 (100)	0	0	34 (94,5)	2 (5,5)	0
EF	32 (100)	0	0	32 (100)	0	0	20 (62,5)	10 (31,25)	2 (6,25)
**Marginal discoloration**									
CSC	35 (100)	0	0	35 (100)	0	0	35 (100)	0	0
FBF	36 (100)	0	0	36 (100)	0	0	34 (94,5)	2 (5,5)	0
EF	32 (100)	0	0	32 (100)	0	0	31 (96,9)	1 (3,1)	0
**Secondary caries**									
CSC	35 (100)	–	0	35 (100)	–	0	35 (100)	–	0
FBF	36 (100)	–	0	36 (100)	–	0	36 (100)	–	0
EF	32 (100)	–	0	32 (100)	–	0	32 (100)	–	0
**Color match**									
CSC	35 (100)	0	0	35 (100)	0	0	35 (100)	0	0
FBF	36 (100)	0	0	36 (100)	0	0	35 (97,2)	1 (2,8)	0
EF	0	6 (19)	26 (81)	0	6 (19)	26 (81)	0	6 (19)	26 (81)
**Postoperative sensitivity**									
CSC	35 (100)	0	0	35 (100)	0	0	35 (100)	0	0
FBF	36 (100)	0	0	36 (100)	0	0	36 (100)	0	0
EF	32 (100)	0	0	32 (100)	0	0	32 (100)	0	0
**Retention**									
CSC	35 (100)	–	0	35 (100)	–	0	35 (100)	–	0
FBF	36 (100)	–	0	36 (100)	–	0	36 (100)	–	0
EF	32 (100)	–	0	32 (100)	–	0	24 (75)	–	8 (25)
**Surface texture**									
CSC	35 (100)	0	0	35 (100)	0	0	35 (100)	0	0
FBF	36 (100)	0	0	36 (100)	0	0	36 (100)	0	0
EF	32 (100)	0	0	32 (100)	0	0	22 (68,8)	9 (28,1)	1 (3,1)

CSC; Charisma Smart Composite, FBF; Filtek Bulkfill Posterior Restorative, EF; Equia Forte Fil A; Alpha B; Bravo C; Charlie

At baseline evaluation, all CSC, FBF and EF restorations were scored as “Alpha” for all criteria except color match. In the EF group, although the most appropriate material color was selected, six restorations were scored as “Bravo” and 26 restorations scored as “Charlie” for color match due to lack of translucency. At the 6-month evaluation, when compared to the baseline evaluation, no significant change was observed in all groups for all criteria (p>0.05).

After one year, survival rates of the CSC and FBF groups were 100%, whereas the survival rate in the EF group was approximately 69%. Ten EF restorations had to be replaced or modified as base because of marginal fracture and material loss in the proximal area at one year.

At one-year evaluation, regarding anatomic form, all restorations in the CSC and FBF groups were scored as “Alpha”. In the EF group, 26 restorations were scored as “Alpha”; four restorations were scored as “Bravo” and two restorations as “Charlie”. For anatomic form criteria, statistically significant difference was found between the EF group and CSC group and between the EF and FBF groups (p<0.05), whereas there was no statistically significant difference between the CSC and FBF groups (p>0.05).

**Figure 4:**
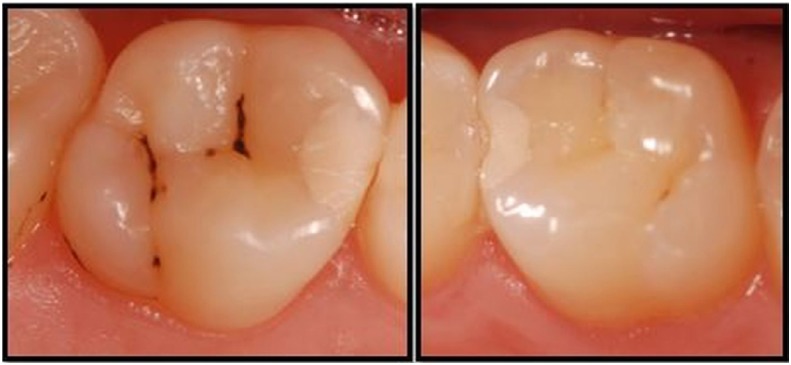
Restorations scored as “Charlie” because of marginal fracture in the EF group at one-year evaluation

At one-year evaluation, regarding contact point criteria, all restorations of the CSC and FBF groups scored as “Alpha”. In the EF group, 27 of the 32 restorations scored as “Alpha” and five restorations (four molar restorations and one premolar restoration) scored as “Charlie” because of marginal fracture (Figure 4). For contact point criteria, there was statistically significant difference between the EF and CSC groups, and between the EF group and FBF group (p<0.05), whereas there was no statistically significant difference between the CSC and FBF groups (p>0.05).

At one-year evaluation, in marginal adaptation criteria, 34 FBF restorations were scored as “Alpha”, two FBF restorations were scored as “Bravo”; 30 CSC restorations were scored as “Alpha” and five CSC restorations were scored as “Bravo”; in the EF group, 20 restorations were evaluated as “Alpha”, 10 restorations as “Bravo” and two as “Charlie. For marginal adaptation criteria, there was statistically significant difference between the EF group and CSC group, and between the EF and FBF groups (p<0.05), whereas there was no statistically significant difference between the CSC and FBF groups (p>0.05).

**Figure 5:**
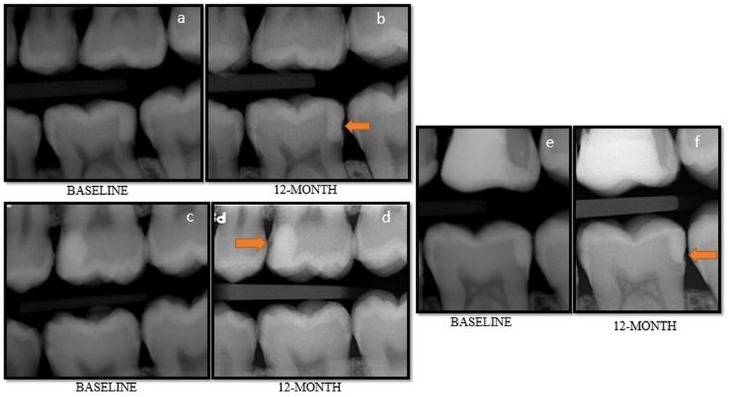
Glass ionomer material loss that could be radiographically seen in the proximal area as a result of dissolution

At one-year evaluation, in retention criteria, all CSC and FBF restorations were scored as “Alpha”. In the EF group, eight restorations were scored as “Charlie” due to marginal fracture and glass ionomer material loss, which could be radiographically seen in the proximal area as a result of dissolution (Figure 5). However, total loss of restorative material was not observed in any of the restorations at the one-year evaluation. For retention criteria, there was statistically significant difference between the EF group and CSC group, and between the EF group and FBF group (p<0.05), whereas there was no statistically significant difference between the CSC and FBF groups (p>0.05).

**Figure 6:**
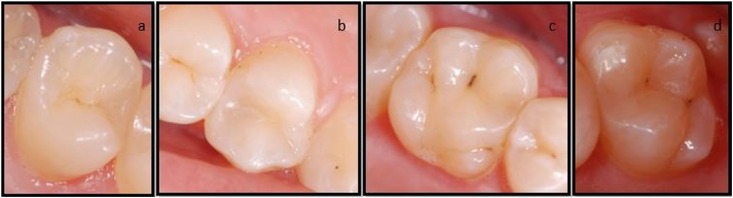
a,b) Restorations scored as “Alpha” for all criteria in the FBF group at one-year evaluation. c,d) Restorations scored as “Alpha” for all criteria in the CSC group at one-year evaluation

**Figure 7:**
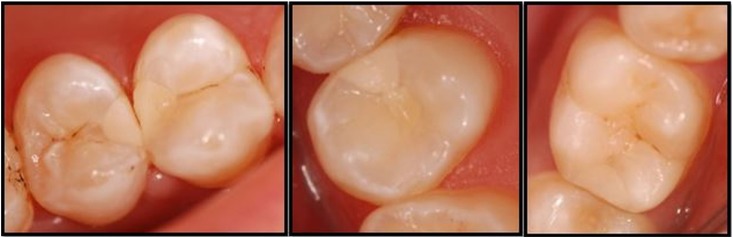
Clinical appearance of some EF restorations at one-year evaluation

At one-year evaluation, no color change occurred in the CSC group, whereas one restoration in the FBF group was evaluated as “Bravo” in terms of color match. In the EF group, no color change was observed between evaluation periods. In term of surface texture, there was no change in the FBF and CSC groups, whereas nine restorations in the EF group were scored as “Bravo” and one restoration was scored as “Charlie” for surface texture. For color match and surface texture criteria, there was statistically significant difference between the EF and CSC groups, and between the EF group and FBF group (p<0.05), whereas there was no statistically significant difference between the CSC and FBF groups (Figures 6, 7) (p>0.05).

Regarding the marginal discoloration criterion, only two FBF restorations and one EF restoration were scored as “Bravo”, whereas other restorations were scored as “Alpha”. For marginal discoloration criteria, there was no statistically significant difference between the groups (p>0.05). During the one-year evaluation, no postoperative sensitivity and secondary caries were observed in any of the restored teeth and all 103 restorations were scored as Alpha (p>0.05).

In this study, ten EF restorations needed replacement at the end of one year. Distribution of failed restorations according to gender, age and type of teeth are shown Table 2.

**Table 2:** Distribution of failed restorations according to gender, age and type of teeth

	UPM (A)	UM (A)	LPM (A)	LM (A)
Female	1 (21)	1 (22)	0	1 (22)
	1 (22)			1 (22)
				1 (22)
Male	1 (22)	1 (22)	0	1 (22)
				1 (22)

UPM: Upper premolar; UM: Upper molar; LPM: Lower premolar; LM: Lower molar; A: Age

## Discussion

In this clinical study, we aimed to evaluate the clinical performances of a micro-hybrid composite resin, a bulk-fill composite resin and a HVGIC in Class II cavities. The clinical performances of the tested materials were evaluated in terms of retention, color match, marginal discoloration, anatomic form, contact point, marginal adaptation, secondary caries, postoperative sensitivity and surface texture. The null hypothesis of this study was rejected because composite resin materials (conventional and bulk-fill) showed better clinical performance than HVGIC.

*In vitro* studies contribute to the development and initial evaluation of restorative materials. Although an attempt is made to imitate clinical conditions, this does not accurately reflect the clinical performance of the materials due to variable parameters into the mouth. Therefore, well-planned, randomized controlled clinical trials are essential to evaluate the clinical performance of newly produced materials and to compare different restorative materials.^17^

Clinical longevity of dental restorations depends on many variables such as the bonding capacity of the restorative material, the application and polymerization technique, the size and shape of the restoration, material-handling skills of dentists and some patient-dependent variables (occlusal forces, intraoral temperature and pH changes).^18^

The HVGIC used in this study is in encapsulated form, which simplifies transporting the material to the cavity and does not require manual mixing. On the other hand, because it can adhere to the handpiece, manipulation and handling of glass ionomer material are harder when compared to composite resin. Nevertheless, in this study, all restorative procedures were performed by a single operator who had advanced clinical training in operative dentistry (at a university) to allow a more-controlled comparison of materials and to avoid any differences between operators. Patients who did not have participation conditions were excluded of the study, and the restorations were randomly made to Class II caries lesions with similar depth.

In this study, cotton pellets and suctions were used to isolate the operative field. The safest way to maintain optimal moisture control is using a rubber dam; however, this is usually impractical in routine activity since it may disturb the patient, and sometimes the placement of the clamp can be traumatic for gingival tissues. Moreover, there are studies in the literature that showed that the use of rubber dam did not affect the clinical behavior of the materials, and that careful isolation with cotton rolls gives similar retention results.^19^,^20^

Objective and reliable criteria for clinical trials should be used to determine the clinical performance of restorative materials. The USPHS criteria are often preferred in clinical follow-up studies and provide ease of direct application to study.^21^,^22^ For this reason, this clinical study evaluated the clinical performance of tested restorative materials using a modified USPHS criteria under the supervision of two different experienced dentists.

Universal adhesives are the latest generation adhesive systems that can be used in both etch & rinse and self-etch modes. In literature, studies report that there is no difference between the application strategies on clinical behavior of universal adhesives.^23^,^24^ However, Marchesi, et al.^25^ (2014) investigated adhesive stability over time of a universal adhesive applied using different bonding techniques on human coronal dentine. They concluded that improved bonding effectiveness of the tested universal adhesive system on dentine was obtained when the adhesive was applied with the self-etch approach. Furthermore, Single Bond Universal adhesive (SBU) contains Vitrebond (3M ESPE), a polyalkenoic acid copolymer that provides chemical bonding with hydroxyapatite crystals, and the high bond strength of SBU is considered to be associated with the polyalkenoic acid copolymer present in its content.^26^–^28^ In this study, SBU was actively applied in self-etch mode (rubbing with a microbrush) to eliminate problems arising from the etching since such procedure is a step that requires a sensitive technique.

Recently, bulk-fill restorative materials such as bulk-fill composite resins and high viscous glass ionomer cements have become very popular materials in operative dentistry because their application is easy and time-saving. With bulk application of restorative materials, some of the challenges have been overcome, such as void formation and contamination risk between the layers, as well as difficulty in the placement of layers in small cavities.

The studies by Çolak, et al.^29^ (2017) and Bayraktar, et al.^30^ (2017) evaluated one-year clinical performance of Class II restorations made using either bulk-fill composite resins or incrementally placed composite resins. These authors reported that both bulk-fill composite resins and incrementally placed composite resin showed comparable and acceptable clinical performance. Similarly, in another study comparing the clinical performances of a nanofill composite resin and a bulk-fill composite resin in Class II cavities, both materials showed similar clinical performance for all criteria up to 12 months. However, at 36-month recall, bulk-fill composite resin demonstrated better clinical performance in terms of marginal discoloration and marginal adaptation, whereas there was no difference between the materials in other parameters.^31^ The 12-month findings of these studies are consistent with our short-term data. In our study, the recall rate was 95% at the end of one year and the survival rate in the FBF and CSC groups was 100%. Furthermore, the bulk-fill composite resin and micro hybrid composite resin showed similar clinical performance, and both materials were found to be clinically successful. These successful results are consistent with the favorable properties of bulk-fill composites mentioned previously. Moreover, long term follow-up is needed to make further comparisons.

Diem, et al.^32^ (2014) evaluated the clinical performance of the Equia restorative system with or without a nanofilled resin coating, comparing micro hybrid composite resin in moderate-depth occlusal cavities on the first permanent molars of 11- to 12-year-old children. They concluded that the Equia System showed an acceptable clinical performance in both conditions.

Gürgan, et al.^16^ (2017) investigated the long-term clinical performance of the Equia restorative system on permanent posterior teeth in Class I and Class II caries lesions according to the USPHS criteria and compared it with a micro hybrid composite resin. The researchers reported that two Equia restorations had to be replaced at three and four years and the cause of failure was mostly due to marginal fracture of restoration. Both restorative materials showed a clinically successful performance after 6 years.

A study conducted by Tal, et al.^33^ (2017) evaluated the clinical and radiographic performances of class II restorations applied HVGIC in primary molars, and it was reported that concavity was radiographically seen on the proximal wall of restorations in 27% of restorations at 18-month recall. The authors also concluded that this material may be effective for Class II restorations in primary molars that are a year or two from shedding. Another study by Scholtanus & Huysmans^34^ reported progressive loss of material in proximal areas of Class II fillings made using HVGIC with coating just below contact areas, being observed on radiographs after 18 months.

Similarly, in our study, five EF restorations had radiographically observable material loss at restoration interface at the end of one year, while there was no change at a six-month evaluation in EF restorations. In addition, at one-year evaluation, 10 Equia restorations were replaced or modified as base under the composite resin, because marginal fracture or glass ionomer material loss on the proximal region resulted food impaction.

The first explanation of this glass ionomer material loss in the proximal area may be related to protective resin. It is very difficult to apply the resin coating to the proximal wall of glass ionomer restoration effectively because the proximal area is not easily accessible. If the surface-coating agent cannot be applied effectively, the proximal area is unprotected from moisture contamination during the initial hardening phase and the glass ionomer cement may dissolve.^33^–^35^

Another explanation of this glass ionomer material loss in the proximal area is the use of metal matrices during restorative procedure. Glass ionomers can chemically adhere to metals, and micro cracks may occur in the glass ionomer cement with the force applied during removal of the matrix. These micro cracks may make the material more susceptible to chemical attacks.^34^

In this study, there was a higher relative risk of failures in molar teeth compared to premolar teeth in EF group when ten failed restorations were analyzed according to tooth location. Moreover, five of these ten restorations were in lower molar teeth. These findings can be explained by the knowledge that restorations of molar teeth are subjected to higher masticatory stresses than restorations of premolar teeth. In addition, chewing forces are strong in lower molar teeth and the increased stress could cause fatigue and fracture of the material, as a result of the position of the lower molar teeth in the dental arch depending on von Spee's curve.^18^,^36^

Furthermore, it has also been reported that surface-coating agents wear over time.^37^ In our study, a slight increase in surface roughness was observed due to the wear of the surface-coating agent at the 12-month evaluation, whereas no surface changes were observed at the 6-month evaluation of EF restorations.

In this study, another problem with EF restorations was color match with the surrounding dental tissue. HVGICs have more translucency than conventional GICs and HVGIC also has more color options. Even so, color and translucency properties of HVGIC restorations were still not enough and its color match was not as good as composite resin restorations during follow-up period in this study. In our study, since the restorations were in the posterior region and the patients were not disturbed by their appearance, the replacement due to color mismatch was not considered. Diem, et al.^32^ (2014) reported that the color match of HVGIC restorations improved over the 3 years of the study (about 25% ‘good’ at baseline, steadily increasing to about 80% ‘good’ at 3 years) with improving translucency over time as the cement matures. In our study, no change in the color match of the restorations was observed between the evaluation periods. However, the duration of our study is one year, and this may not be enough for exact cement maturation.

In literature, although clinical studies^16^,^38^,^39^ with very low failure rate are available, the high failure rate (31%) was shown for HVGIC after 12 months in our study. Menezes-Silva, et al.^38^ (2016) reported that excellent success rates were shown, and the authors attributed the high success rate in their study to the fact that most of the cavities were relatively small and that they prepared additional retentions in proximal boxes. Whereas in our study the cavity size was mostly moderate and additional retention was not prepared. Furthermore, the authors in the studies with very low failure rates evaluated Equia Fil as a HVGIC, whereas we evaluated the clinical performance of Equia Forte Fil. Although both materials are high viscosity glass ionomers, their ingredients are not completely the same. Equia Forte Fil's powder includes additionally higher molecular weight polyacrylic acid and highly reactive small particles. Moreover, the evaluation may affect the study results as well as the variables of patients or operators. Although the same criteria were used in studies, the evaluators could apply the evaluation criteria more subjectively. For these reasons, it may be inaccurate to make direct comparisons with previous studies.

Glass ionomer cements could be used as a semi-permanent restorative material for patients with a high caries activity to control the disease by releasing fluoride. Rapid caries removal and temporization eliminates the infection as quickly as possible. Moreover, this method allows for a more accurate assessment of restorability and prognosis for each individual tooth. For deep caries lesions, stepwise caries removal could also be an option to avoid pulpal complications during disease control. In this treatment protocol, after 6-8 months, temporarily treated teeth are re-entered, all remaining demineralized dentin is removed, and a final treatment is provided as appropriate.^40^ In this study, it was found that high viscosity glass ionomer material was unsuitable as permanent restorative material within the tested situations. However, the use of this glass ionomer material as semi-permanent material can be considered.

Short evaluation time is one of the limitations of this study. Although long-term follow-up is important to compare and evaluate the clinical performances of restorative materials, short-term clinical data can also give some useful information about the clinical performances of the materials. Although our study is a short-term clinical study, the patients will continue to be followed-up for additional evaluations.

The fact that it is not designed as split-mouth is another limitation of this study. Split-mouth study designs can reduce most inter-patient variability such as oral hygiene, diet and brushing habits etc. on the longevity of restorations. The possible patient loss is a disadvantage of split-mouth designs since more restorations than one would be lost when a patient did not come for follow-up appointment. Although this study was not designed as split-mouth and the variables between patients were ignored, the patients not fulfilling the inclusion criteria were excluded in the study.

## Conclusion

At the end of one year, both highly viscous bulk-fill composite resin and conventional micro hybrid composite resin showed similar and successful clinical performance whereas HVGIC showed worse. However, the use of high viscosity glass ionomer material as a semi-permanent restorative material in stress bearing Class II cavities rather than permanent material might be more appropriate since high failure rates were observed after one year.
